# Supplementary material for: The impact of Evidence-Based Pharmacy on the quality of pharmaceutical care: A survey study
Source: PLoS One. 2025 Aug 1;20(8):e0329016. doi: 10.1371/journal.pone.0329016 (PMC12316249; doi:10.1371/journal.pone.0329016)
Supplement: S1 Table — (DOCX) [file pone.0329016.s001.docx]

S1 Table. Statistical analysis – additional results (correlations between questions).

| **Question** | | | | **Question VERSUS question (section number/question number)** | **Correlation** | **Interpretation** |
| --- | --- | --- | --- | --- | --- | --- |
| **How would you rate your knowledge of Evidence-Based Pharmacy?** | | | | 11 vs 4  Chi^2^=33.90973  df=12  p=0.00070  CramersV= 0.136 (0.097 – 0.175) | EBPharm knowledge level vs education level | Respondents with a bachelor’s or master’s degree rate their knowledge higher than pharmacy technicians. |
|  |  |  |  | 11 vs 5  Chi^2^=67.09576  df=36  p=0.00126  CramersV= 0.271 (0.221 – 0.328) | EBPharm knowledge level vs job position | Pharmacists working in community pharmacies rate their knowledge higher than pharmacy technicians. |
| **Do you think Evidence-Based Pharmacy influences/could influence the quality of pharmaceutical care and increases patient safety?** | | | | 13 vs 2  Chi^2^=12.74131  df=6  p=0.04733  CramersV= 0.166 (0.077 – 0.270) | EBPharm influence vs age | Regardless of age, respondents believe EBPharm influences the quality of care and patient safety. |
|  |  |  |  | 13 vs 4  Chi^2^=21.60257  df=6  p=0.00143  CramersV= 0.135 (0.086 – 0.191) | EBPharm influence vs education level | Regardless of education level, respondents believe that EBPharm influences the quality of care and patient safety. |
|  |  |  |  | 13 vs 6  Chi^2^=16.49449  df=8  p=0.03583  CramersV= 0.172 (0.101 – 0.241) | EBPharm influence vs salary level | Regardless of income, respondents believe EBPharm influences the quality of care and patient safety. |
|  |  |  |  | 13 vs 5  Chi^2^=45.07867  df=9  p=0.00000  CramersV= 0.213 (0.136 – 0.287) | EBPharm influence vs job position | Regardless of job position, respondents believe EBPharm influences the quality of care and patient safety. |
|  |  |  |  | 13 vs 7  Chi^2^=17.91459  df=6  p=0.00645  CramersV= 0.184 (0.113 – 0.259) | EBPharm influence vs work experience | Regardless of work experience, respondents believe that EBPharm influences the quality of care and patient safety. |
| **Do you feel the need for further education in Evidence-Based Pharmacy?** | | | | 14 vs 10  Chi^2^=27.40076  df=1  p=0.00000  CramersV= 0.235 (0.000 – 0.496) | Need of further EBPharm education vs applying EBPharm principles | Respondents applying EBPharm in their daily practice feel the need for further education in this area. |
| **Do you check the reliability of the sources you read?** | | | | 15 vs 1  Chi^2^=10.90937  df=2  p=0.00428  CramersV= 0.209 (0.131 – 0.302) | Checking of source reliability vs gender | Men are more likely to check the reliability of data sources. |
|  |  |  |  | 15 vs 4  Chi^2^=28.31064  df=6  p=0.00008  CramersV= 0.129 (0.072 – 0.198) | Checking of source reliability vs education level | Respondents with a bachelor’s or master’s degree are more likely to verify the reliability of sources than pharmacy technicians. |
|  |  |  |  | 15 vs 5  Chi^2^=30.14473  df=18  p=0.03606  CramersV= 0.242 (0.187 – 0.296) | Checking of source reliability vs job position | Pharmacists working in community pharmacies check the reliability of sources more often than pharmacy technicians. |
|  |  |  |  | 15 vs 10  Chi^2^=22.45923  df=2  p=0.00001  CramersV= 0.324 (0.230 – 0.479) | Checking of source reliability vs applying EBPharm principles | Those who apply EBPharm principles check the reliability of sources. |
| **Do you regularly update your knowledge using the latest scientific publications?** | | | | 16 vs 1  Chi^2^=7.437519  df=2  p=0.02426  CramersV= 0.171 (0.043 – 0.286) | Updating the knowledge vs gender | Respondents, regardless of gender, rarely update their knowledge. |
|  |  |  |  | 16 vs 2  Chi^2^=14.28446  df=6  p=0.02662  CramersV= 0.187 (0.070 – 0.364) | Updating the knowledge vs age | Regardless of age, respondents occasionally update their knowledge. |
|  |  |  |  | 16 vs 6  Chi^2^=15.59294  df=8  p=0.04859  CramersV= 0.175 (0.109 – 0.239) | Updating the knowledge vs salary level | Knowledge is rarely updated by those earning between 2,500 and 7,000 PLN (1160-1630 €). |
| **Which of the following scientific information sources do you use in your work?** | | | Publications in scientific journals | 17 vs 2  Chi^2^=7.883308  df=3  p=0.04849  CramersV= 0.180 (0.080 – 0.292) | Source of information vs age | Regardless of age, respondents use scientific journal publications in their work. |
|  |  |  |  | 17 vs 4  Chi^2^=13.92150  df=3  p=0.00301  CramersV= 0.173 (0.091 – 0.255) | Source of information vs education level | Regardless of education level, respondents use scientific journal publications in their work. |
|  |  |  |  | 17 vs 10  Chi^2^=7.459799  df=1  p=0.00631  CramersV= 0.132 (0.000 – 0.264) | Source of information vs applying EBPharm principles | Those who apply EBPharm principles are more likely to read scientific journal publications. |
|  |  |  | Conference reports | 17 vs 1  Chi^2^=5.365187  df=1  p=0.02054  CramersV= 0.125 (0.023 – 0.227) | Source of information vs gender | Conference reports are used regardless of gender. |
|  |  |  |  | 17 vs 4  Chi^2^=31.22887  df=3  p=0.00000  CramersV= 0.149 (0.066 – 0.245) | Source of information vs education level | The higher the education level, the more often conference reports are used. |
|  |  |  |  | 17 vs 5  Chi^2^=33.85540  df=9  p=0.00009  CramersV= 0.348 (0.277 – 0.431) | Source of information vs job position | Pharmacists in community pharmacies use conference reports more often than pharmacy technicians. |
|  |  |  |  | 17 vs 6  Chi^2^=25.44861  df=4  p=0.00004  CramersV= 0.302 (0.201 – 0.395) | Source of information vs salary level | The higher the salary level, the more frequently conference reports are used. |
|  |  |  |  | 17 vs 10  Chi^2^=11.35537  df=1  p=0.00075  CramersV= 0.175 (0.062 – 0.273) | Source of information vs applying EBPharm principles | Those applying EBPharm principles read conference reports more often. |
|  |  |  | The Internet (forums, blogs, social media) | 17 vs 1  Chi^2^=3.992347  df=1  p=0.04571  CramersV= 0.106 (0.001 – 0.221) | Source of information vs gender | Women use the Internet more often. |
|  |  |  |  | 17 vs 5  Chi^2^=30.74980  df=9  p=0.00033  CramersV= 0.340 (0.264 – 0.413) | Source of information vs job position | Pharmacy technicians use the Internet more frequently than pharmacists in community pharmacies. |
|  |  |  |  | 17 vs 6  Chi^2^=24.85063  df=4  p=0.00005  CramersV= 0.295 (0.198 – 0.384) | Source of information vs salary level | The Internet is more frequently used by those earning between 2,500 and 5,000 PLN (1160-1630 €). |
|  |  |  | Academic books | 17 vs 4  Chi^2^=36.41106  df=3  p=0.00000  CramersV= 0.170 (0.071 – 0.264) | Source of information vs education level | Pharmacy technicians less frequently use academic books. |
|  |  |  |  | 17 vs 5  Chi^2^=38.33240  df=9  p=0.00002  CramersV= 0.371 (0.289 – 0.466) | Source of information vs job position | Pharmacists in community pharmacies use books more frequently than pharmacy technicians. |
|  |  |  |  | 17 vs 6  Chi^2^=16.60777  df=4  p=0.00230  CramersV= 0.247 (0.145 – 0.334) | Source of information vs salary level | The higher the salary level, the more frequently academic books are used. |
|  |  |  |  | 17 vs 7  Chi^2^=8.312285  df=3  p=0.03998  CramersV= 0.183 (0.087 – 0.281) | Source of information vs work experience | The longer the professional experience, the less interest in books. |
|  |  |  | Professional training/courses | 17 vs 4  Chi^2^=23.30309  df=3  p=0.00003  CramersV= 0.206 (0.137 – 0.288) | Source of information vs education level | The higher the education level, the less interest in professional training and courses. |
|  |  |  |  | 17 vs 5  Chi^2^=29.98466  df=9  p=0.00044  CramersV= 0.337 (0.253 – 0.419) | Source of information vs job position | Pharmacy technicians use training and courses more frequently than pharmacists in community pharmacies. |
|  |  |  |  | 17 vs 10  Chi^2^=3.900444  df=1  p=0.04827  CramersV= 0.095 (0.000 – 0.201) | Source of information vs applying EBPharm principles | Those applying EBPharm principles are less likely to use training/courses. |
|  |  |  | Webinars | 17 vs 4  Chi^2^=13.38482  df=3  p=0.00387  CramersV= 0.177 (0.085 – 0.283) | Source of information vs education level | Students use webinars more frequently than individuals with a bachelor’s or master’s degree or pharmacy technicians. |
|  |  |  |  | 17 vs 6  Chi^2^=10.35310  df=4  p=0.03488  CramersV= 0.205 (0.112 – 0.303) | Source of information vs salary level | Interest in webinars decreases with salary level. |
|  |  |  |  | 17 vs 7  Chi^2^=12.58718  df=3  p=0.00562  CramersV= 0.216 (0.119 – 0.308) | Source of information vs work experience | Interest in webinars decreases with professional experience. |
| **Are you able to assess the level of scientific evidence in pharmacy-related research articles?** | | | | 18 vs 1  Chi^2^=6.357902  df=2  p=0.04163  CramersV= 0.145 (0.057 – 0.243) | Assessment of evidence vs gender | Respondents, regardless of gender, can assess the level of scientific evidence. |
|  |  |  |  | 18 vs 4  Chi^2^=45.01363  df=6  p=0.00000  CramersV= 0.118 (0.061 – 0.176) | Assessment of evidence vs education level | Respondents with a bachelor’s or master’s degree can assess the level of scientific evidence. |
|  |  |  |  | 18 vs 5  Chi^2^=45.79460  df=18  p=0.00032  CramersV= 0.299 (0.236 – 0.391) | Assessment of evidence vs job position | Pharmacists working in community pharmacies have higher qualifications in this area than pharmacy technicians. |
| **Do you know the PICO framework (Population, Intervention, Comparison, Outcome) – a tool for evaluating the quality of information?** | | | | 19 vs 2  Chi^2^=9.273501  df=3  p=0.02587  CramersV= 0.189 (0.084 – 0.305) | Knowing PICO vs age | Respondents, regardless of age, do not know the PICO framework. |
|  |  |  |  | 19 vs 7  Chi^2^=16.71975  df=3  p=0.00081  CramersV= 0.241 (0.145 – 0.331) | Knowing PICO vs work experience | Knowledge of the PICO framework decreases with professional experience. |
|  |  |  |  | 19 vs 10  Chi^2^=5.500680  df=1  p=0.01901  CramersV= 0.113 (0.071 – 0.153) | Knowing PICO vs applying EBPharm principles | Those applying EBPharm principles know the PICO framework. |
|  |  |  |  | 19 vs 12  Chi^2^=59.21981  df=1  p=0.00000  CramersV= 0.429 (0.320 – 0.533) | Knowing PICO vs EBPharm training | Those who have completed EBPharm training know and use the PICO framework. |
| **Do you use scientific evidence such as research publications in your work?** | | | | 20 vs 2  Chi^2^=24.41822  df=6  p=0.00044  CramersV= 0.222 (0.104 – 0.363) | Using scientific evidence vs age | Respondents, regardless of age, rarely use scientific evidence. |
|  |  |  |  | 20 vs 5  Chi^2^=38.40711  df=18  p=0.00342  CramersV= 0.283 (0.225 – 0.341) | Using scientific evidence vs job position | Pharmacists in community pharmacies use scientific evidence much more often than pharmacy technicians. |
|  |  |  |  | 20 vs 6  Chi^2^=26.98435  df=8  p=0.00071  CramersV= 0.227 (0.166 – 0.289) | Using scientific evidence vs salary level | The frequency of using scientific evidence increases with salary level. |
|  |  |  |  | 20 vs 7  Chi^2^=19.22415  df=6  p=0.00380  CramersV= 0.199 (0.117 – 0.278) | Using scientific evidence vs work experience | The frequency of using scientific evidence decreases with professional experience. |
|  |  |  |  | 20 vs 10  Chi^2^=32.91160  df=2  p=0.00000  CramersV= 0.322 (0.120 – 0.546) | Using scientific evidence vs applying EBPharm principles | Those familiar with EBPharm principles frequently use scientific evidence. |
| **Is the affiliation (institutional association) of the authors of a scientific paper and funding information important for your analysis of the paper?** | | | | 20.1. vs 6  Chi^2^=21.41978  df=4  p=0.00026 | Importance of affiliation and funding information vs | Regardless of salary level, respondents consider information on affiliation and funding important. |
|  |  |  |  | 20.1 vs 10  Chi^2^=5.439155  df=1  p=0.01969 | Importance of affiliation and funding information vs applying EBPharm principles | Those familiar with EBPharm principles consider affiliation and funding information important. |
| **What criteria do you consider when selecting a scientific publication?** | | Publication date | | 20.2 vs 2  Chi^2^=18.11619  df=3  p=0.00042  CramersV= 0.254 (0.102 – 0.405) | Criterium of selection vs age | Regardless of age, respondents consider the publication date when selecting a scientific publication. |
|  |  |  |  | 20.2 vs 6  Chi^2^=16.61908  df=4  p=0.00229  CramersV= 0.304 (0.216 – 0.452) | Criterium of selection vs salary level | The importance of the publication date increases with salary level. |
|  |  |  |  | 20.2 vs 15  Chi^2^=6.127496  df=2  p=0.04671 | Criterium of selection vs checking source reliability | Those checking the reliability of data sources consider the publication date. |
|  |  | Funding information | | 20.2 vs 10  Chi^2^=6.453092  df=1  p=0.01108  CramersV= 0.035 (0.000 – 0.115) | Criterium of selection vs applying EBPharm principles | Those applying EBPharm principles more frequently consider funding information. |
|  |  |  |  | 20.2. vs 15  Chi^2^=9.506703  df=2  p=0.00862 | Criterium of selection vs checking source reliability | Funding information is primarily considered by those who always check the reliability of the sources they read. |
|  |  | Accessibility of the publication in databases | | 20.2. vs 4  Chi^2^=18.56735  df=3  p=0.00034  CramersV= 0.134 (0.053 – 0.226) | Criterium of selection vs education level | The availability of publications in databases is checked more frequently by individuals with bachelor’s or master’s degrees than pharmacy technicians. |
|  |  |  |  | 20.2. vs 5  Chi^2^=25.48552  df=9  p=0.00248  CramersV= 0.319 (0.243 – 0.415) | Criterium of selection vs job position | Pharmacists in community pharmacies check database availability much more often than pharmacy technicians. |
|  |  |  |  | 20.2. vs 6  Chi^2^=15.34627  df=4  p=0.00403  CramersV= 0.246 (0.157 – 0.348) | Criterium of selection vs salary level | The higher the salary, the more frequently respondents check the availability of publications in databases. |
|  |  |  |  | 20.2. vs 18  Chi^2^=11.88160  df=2  p=0.00263  CramersV= 0.199 (0.106 – 0.302) | Criterium of selection vs assessment of evidence level | Database availability is checked more frequently by those able to assess the level of scientific evidence. |
|  |  | Research methodology | | 20.2 vs 4  Chi^2^=14.41574  df=3  p=0.00239  CramersV= 0.168 (0.078 – 0.267) | Criterium of selection vs education level | Research methodology is more often verified by those with a bachelor’s or master’s degrees than pharmacy technicians. |
|  |  |  |  | 20.2 vs 5  Chi^2^=27.91296  df=9  p=0.00099  CramersV= 0.323 (0.246 – 0.399) | Criterium of selection vs job position | Research methodology is more frequently verified by pharmacists working in community pharmacies than by pharmacy technicians. |
|  |  |  |  | 20.2. vs 6  Chi^2^=10.86258  df=4  p=0.02815  CramersV= 0.217 (0.124 – 0.312) | Criterium of selection vs salary level | The importance of methodology as a section of scientific publications increases with salary. |
|  |  |  |  | 20.2. vs 15  Chi^2^=21.99548  df=2  p=0.00002 | Criterium of selection vs checking source reliability | The methodology is more frequently verified by those who check the reliability of the sources they read. |
|  |  |  |  | 20.2 vs 18  Chi^2^=14.90177  df=2  p=0.00058  CramersV= 0.233 (0.128 – 0.340) | Criterium of selection vs assessment of evidence level | Methodology is often verified by those capable of assessing the level of evidence. |
|  |  | Information on conflicts of interest | | 20.2 vs 2  Chi^2^=12.52998  df=3  p=0.00577 | Criterium of selection vs age | The frequency of verifying conflicts of interest decreases with age. |
| **How often do you use medical databases in your pharmaceutical practice or studies?** | | | | 21 vs 4  Chi^2^=81.66593  df=12  p=0.00000  CramersV= 0.184 (0.135 – 0.259) | Using medical databases vs education level | Those with a bachelor’s or master’s degree use medical databases more often than pharmacy technicians. |
|  |  |  |  | 21 vs 5  Chi^2^=244.7647  df=36  p=0.00000  CramersV= 0.507 (0.407 – 0.634) | Using medical databases vs job position | Pharmacists in community pharmacies use medical databases more often than pharmacy technicians. |
| **Please indicate the medical databases you use.** | | PubMed | | 21.1 vs 16  Chi^2^=9.020642  df=2  p=0.01099  CramersV= 0.211 (0.018 – 0.464) | Using specific database vs knowledge update | For those who regularly update their knowledge, PubMed is the first choice among medical databases. |
|  |  | Cochrane Library | | 21.1 vs 1  Chi^2^=4.747778  df=1  p=0.02934  CramersV= 0.124 (0.002 – 0.246) | Using specific database vs gender | Men use the Cochrane Library more often. |
|  |  |  |  | 21.1 vs 4  Chi^2^=14.98630  df=3  p=0.00183  CramersV= 0.141 (0.063 – 0.221) | Using specific databas vs education level | The Cochrane Library is more often used by those with a bachelor’s or master’s degrees than pharmacy technicians. |
|  |  |  |  | 21.1 vs 5  Chi^2^=20.45557  df=9  p=0.01530  CramersV= 0.310 (0.245 – 0.376) | Using specific databas vs job position | The Cochrane Library is also more frequently used by pharmacists in community pharmacies than pharmacy technicians. |
|  |  |  |  | 21.1 vs 6  Chi^2^=10.76426  df=4  p=0.02935 | Using specific databas vs salary level | Interest in the Cochrane Library increases with salary level. |
|  |  | Embase | | 21.1 vs 2  Chi^2^=9.456711  df=3  p=0.02380  CramersV= 0.216 (0.060 – 0.410) | Using specific databas vs age | Regardless of age, respondents do not use Embase. |
|  |  |  |  | 21.1 vs 7  Chi^2^=9.596345  df=3  p=0.02233  CramersV= 0.204 (0.061 – 0.376) | Using specific databas vs work experience | Regardless of work experience, respondents do not use Embase. |
|  |  |  |  | 21.1 vs 12  Chi^2^=20.54185  df=1  p=0.00001  CramersV= 0.255 (0.138 – 0.367) | Using specific databas vs EBPharm training | Embase use is more frequent among those who have undergone EBPharm training. |
|  |  | Web of Science | | 21.1 vs 4  Chi^2^=8.737409  df=3  p=0.03299  CramersV= 0.201 (0.073 – 0.354) | Using specific databas vs education level | People with a bachelor’s or master’s degree use the Web of Science more often. |
|  |  |  |  | 21.1 vs 5  Chi^2^=39.26778  df=9  p=0.00001  CramersV= 0.391 (0.252 – 0.530) | Using specific databas vs job position | Pharmacists in community pharmacies use the Web of Science more often than technicians (while technicians do not use it at all). |
|  |  |  |  | 21.1 vs 6  Chi^2^=18.21679  df=4  p=0.00112 | Using specific databas vs salary level | The higher the salary level, the more frequent the use of Web of Science. |
|  |  |  |  | 21.1 vs 7  Chi^2^=12.39211  df=3  p=0.00615  CramersV= 0.226 (0.082 – 0.393) | Using specific databas vs work experience | Regardless of work experience, respondents rarely use the Web of Science. |
|  |  |  |  | 21.1 vs 12  Chi^2^=6.042370  df=1  p=0.01397  CramersV= 0.133 (0.008 – 0.254) | Using specific databas vs EBPharm training | EBPharm training increases the popularity of the Web of Science. |
|  |  | Scopus | | 21.1 vs 4  Chi^2^=14.94413  df=3  p=0.00187  CramersV= 0.183 (0.065 – 0.320) | Using specific databas vs education level | Those with a bachelor’s or master’s degree use Scopus much more frequently than pharmacy technicians or students (both groups do not use it at all). |
|  |  |  |  | 21.1 vs 5  Chi^2^=80.40624  df=9  p=0.00000  CramersV= 0.580 (0.475 – 0.702) | Using specific databas vs job position | Pharmacists in community pharmacies much more often use Scopus than by other employed groups. |
|  |  |  |  | 21.1 vs 12  Chi^2^=7.572386  df=1  p=0.00593  CramersV= 0.152 (0.031 – 0.270) | Using specific databas vs EBPharm training | Those who have undergone EBPharm training use Scopus much more frequently. |
|  |  |  |  | 21.1 vs 16  Chi^2^=10.59018  df=2  p=0.00502  CramersV= 0.199 (0.088 – 0.313) | Using specific databas vs knowledge update | Those who regularly update their knowledge often use Scopus. |
|  |  | Science Direct | | 21.1 vs 4  Chi^2^=10.88580  df=3  p=0.01236  CramersV= 0.230 (0.091 – 0.385) | Using specific databas vs education level | Science Direct is used more often by people with a bachelor’s or master’s degree. |
|  |  |  |  | 21.1 vs 5  Chi^2^=46.80512  df=9  p=0.00000  CramersV= 0.469 (0.370 – 0.586) | Using specific databas vs job position | Science Direct is used more often by pharmacists in community pharmacies. |
|  |  |  |  | 21.1 vs 12  Chi^2^=7.499002  df=1  p=0.00617  CramersV= 0.152 (0.021 – 0.277) | Using specific databas vs EBPharm training | Those trained in EBPharm use Science Direct much more frequently. |
|  |  |  |  | 21.1 vs 16  Chi^2^=9.812586  df=2  p=0.00740  CramersV= 0.188 (0.068 – 0.309) | Using specific databas vs knowledge update | Those who regularly update their knowledge use Science Direct more frequently. |
| **Are you able to conduct a systematic review of the scientific literature in the field of pharmacy?** | | | | 22 vs 2  Chi^2^=19.17643  df=6  p=0.00388  CramersV= 0.206 (0.133 – 0.298) | Ability to conduct a systematic review vs | People aged <26 (including students and those who have completed courses) are more likely to partially conduct systematic reviews than other age groups. |
|  |  |  |  | 22 vs 4  Chi^2^=59.79156  df=6  p=0.00000  CramersV= 0.160 (0.093 – 0.238) | Ability to conduct a systematic review vs education level | Those with a bachelor’s or master’s degree are partially able to conduct systematic reviews, while pharmacy technicians are not able to conduct them at all. |
|  |  |  |  | 22 vs 5  Chi^2^=144.2861  df=18  p=0.00000  CramersV= 0.519 (0.407 – 0.634) | Ability to conduct a systematic review vs job position | Pharmacists in community pharmacies are partially able to conduct systematic reviews, while most technicians do not have this ability. |
|  |  |  |  | 22 vs 7  Chi^2^=36.64887  df=6  p=0.00000  CramersV= 0.262 (0.163 – 0.381) | Ability to conduct a systematic review vs work experience | The ability to conduct systematic reviews decreases with work experience. |
| **Are you able to assess the quality and reliability of scientific research and pharmacy-related work published in scientific journals and available in medical databases?** | | | | 23 vs 4  Chi^2^=39.80967  df=3  p=0.00000  CramersV= 0.137 (0.058 – 0.228) | Ability to assess the quality and reliability vs education level | Respondents with a bachelor’s or master’s degree are much more often able to assess the quality and reliability of scientific studies than pharmacy technicians. |
|  |  |  |  | 23 vs 5  Chi^2^=39.50271  df=9  p=0.00001  CramersV= 0.370 (0.278 – 0.475) | Ability to assess the quality and reliability vs job position | Pharmacists in community pharmacies are more often able to assess the quality and reliability of scientific studies than pharmacy technicians. |
|  |  |  |  | 23 vs 7  Chi^2^=12.02077  df=3  p=0.00731  CramersV= 0.209 (0.103 – 0.323) | Ability to assess the quality and reliability vs work experience | The ability to assess the quality and reliability of data increases with work experience. |
|  |  |  |  | 23 vs 10  Chi^2^=9.828913  df=1  p=0.00172  CramersV= 0.159 (0.021 – 0.273) | Ability to assess the quality and reliability vs applying EBPharm principles | Those applying EBPharm principles can assess the quality and reliability of data. |
| **How would you rate the reliability of the following types of research?** | Case report | | | 24 vs 10  Chi^2^=4.961071  df=1  p=0.02592  CramersV= 0.102 (0.000 – 0.276) | Source reliability vs applying EBPharm principles | Those applying EBPharm principles more often provide the correct answer regarding case analysis—low reliability. |
|  |  |  |  | 24 vs 18  Chi^2^=6.114999  df=2  p=0.04701  CramersV= 0.163 (0.042 – 0.307) | Source reliability vs assessment of evidence level | Those capable of assessing the level of scientific evidence more often provide the correct answer regarding case analysis (low reliability). |
|  | Case series | | | 24 vs 4  Chi^2^=9.978770  df=3  p=0.01875  CramersV= 0.117 (0.047 – 0.203) | Source reliability vs education level | Respondents with a bachelor’s or master’s degree more frequently than pharmacy technicians or students provided the correct answer regarding case series (low reliability). |
|  |  |  |  | 24 vs 5  Chi^2^=18.09444  df=9  p=0.03409  CramersV= 0.280 (0.228 – 0.337) | Source reliability vs job position | Pharmacists in community pharmacies more frequently than pharmacy technicians provided the correct answer regarding case series (low reliability) |
|  |  |  |  | 24 vs 10  Chi^2^=7.329146  df=1  p=0.00678  CramersV= 0.133 (0.029 – 0.223) | Source reliability vs applying EBPharm principles | Those applying EBPharm principles more frequently provided the correct answer regarding case series (low reliability). |
|  | Case-control studies | | | 24 vs 4  Chi^2^=12.85697  df=6  p=0.04536  CramersV= 0.157 (0.101 – 0.215) | Source reliability vs education level | Students and those with a bachelor’s or master’s degree more frequently gave the correct answer for case-control studies (moderate reliability). |
|  | Meta-analyses | | | 24 vs 10  Chi^2^=8.106696  df=2  p=0.01736  CramersV= 0.188 (0.038 – 0.374) | Source reliability vs applying EBPharm principles | Those applying EBPharm principles more often provided the correct answer regarding meta-analyses (high reliability). |
|  |  |  |  | 24 vs 12  Chi^2^=14.74316  df=2  p=0.00063  CramersV= 0.219 (0.129 – 0.314) | Source reliability vs EBPharm training | Those who have undergone EBPharm training more often provided the correct answer regarding meta-analyses (high reliability). |
|  |  |  |  | 24 vs 18  Chi^2^=15.48424  df=4  p=0.00380  CramersV= 0.167 (0.094 – 0.242) | Source reliability vs assessment of evidence level | Those capable of assessing the level of scientific evidence more frequently provided the correct answer regarding meta-analyses (high reliability). |
| **Are you able to analyse research results and translate them into understandable clinical information for patients or other healthcare professionals?** | | | | 25 vs 4  Chi^2^=27.63732  df=6  p=0.00011  CramersV= 0.149 (0.089 – 0.213) | Ability to analyse and transform the results vs education level | Regardless of education, respondents can analyse research results and transform them into understandable clinical information for patients or other healthcare professionals. |
|  |  |  |  | 25 vs 5  Chi^2^=42.99682  df=18  p=0.00080  CramersV= 0.283 (0.228 – 0.335) | Ability to analyse and transform the results vs job position | Both pharmacists in community pharmacies and pharmacy technicians can analyse and transform research results. |
|  |  |  |  | 25 vs 10  Chi^2^=6.978717  df=2  p=0.03052  CramersV= 0.169 (0.053 – 0.354) | Ability to analyse and transform the results vs applying EBPharm principles | Those applying EBPharm principles can analyse research results and transform them. |
|  |  |  |  | 25 vs 18  Chi^2^=44.26496  df=4  p=0.00000  CramersV= 0.267 (0.212 – 0.317) | Ability to analyse and transform the results vs assessment of evidence level | Those capable of assessing the level of scientific evidence can analyse and transform results. |
