# Supplementary material for: The impact of Evidence-Based Pharmacy on the quality of pharmaceutical care: A survey study
Source: PLoS One. 2025 Aug 1;20(8):e0329016. doi: 10.1371/journal.pone.0329016 (PMC12316249; doi:10.1371/journal.pone.0329016)
Supplement: S1 File — Survey questionnaire. (DOCX) [file pone.0329016.s002.docx]

SURVEY

Dear Sir/Madam,

As a student of pharmacy at the Karol Marcinkowski University of Medical Sciences in Poznan, I kindly request your participation in the following survey, which serves as the primary data source for my master's thesis entitled "Evidence-Based Pharmacy in the Work of Polish Pharmacists."

The survey is aimed at individuals working in pharmacies and at pharmacy students currently undergoing professional internships.

Please ensure that your responses are used solely for academic purposes and that the survey is completely anonymous. Your identities will remain confidential.

Completing the survey took no more than 5-10 minutes.

For more information regarding the survey, please contact the research coordinators Barbara Jacieczko at barbaracieczko@gmail.com and Dr Piotr Ratajczak (supervisor) at p_ratajczak@ump.edu.pl.

1. Gender:
2. Female
3. Male
4. Non-binary
5. Prefer not to say
6. Other: ______________________
7. Age:
8. ≤ 26
9. 26-39
10. 40-59
11. ≥ 60
12. Size of your place of residence:
13. Village
14. Town up to 100,000 residents
15. Town up to 250,000 residents
16. Town up to 500,000 residents
17. City with more than 500,000 residents
18. Your level of education:
19. Primary
20. Pharmacy technician
21. Secondary (or post-secondary)
22. Student
23. Higher education, first cycle (bachelor's or master’s degree)
24. Higher education, second cycle (doctorate)
25. Other: ______________________
26. Your job position (you can select more than one answer):
27. Pharmacist in an open pharmacy
28. Pharmacist in a closed pharmacy
29. Pharmacy technician
30. Clinical research worker
31. Pharmaceutical industry employee
32. Research worker
33. Currently not employed
34. Other: ______________________
35. What is your average net salary?
36. ≤ 2500 PLN (≤ 580 €)
37. 2500-5000 PLN (580-1160 €)
38. 5000-7000 PLN (1160-1630 €)
39. ≥ 7000 PLN (≥ 1630 €)
40. Currently not employed
41. How long have you been working in the profession?
42. 0-5 years
43. 6-10 years
44. 11-19 years
45. ≥20 years
46. Do you have a specialisation in pharmacy?
47. Yes
48. No
    1. If yes, what specialisation do you have? (you can select more than one answer)
49. Clinical Pharmacy
50. Hospital Pharmacy
51. Pharmacology
52. Pharmacoeconomics
53. Toxicology
54. Medical Analytics
55. Herbal Medicine
56. Other: ______________________
57. Do you participate in scientific research or pharmacy-related research projects?
58. Yes
59. No
60. Do you apply the principles of Evidence-Based Pharmacy (based on the concept of Evidence-Based Medicine) in your professional practice?
61. Yes
62. No
63. How would you rate your knowledge of Evidence-Based Pharmacy?
64. Very good
65. Good
66. Average
67. Poor
68. I have no knowledge of it
69. Were you trained in Evidence-Based Pharmacy during your studies or professional practice?
70. Yes
71. No
    1. If yes, was this training sufficient?
    2. Yes
    3. No
72. Do you think Evidence-Based Pharmacy influences/could influence the quality of pharmaceutical care and increases patient safety?
73. Yes
74. No
75. I don’t know
76. Do you feel the need for further education in Evidence-Based Pharmacy?
77. Yes
78. No
79. Do you check the reliability of the sources you read?
80. Yes, always
81. Yes, sometimes
82. No, never
83. Do you regularly update your knowledge using the latest scientific publications?
84. Yes, always
85. Yes, sometimes
86. No, never
87. Which of the following scientific information sources do you use in your work? (you can select more than one answer)
88. Publications in scientific journals
89. Academic books
90. Webinars
91. Conference reports
92. Professional training/courses
93. The internet (forums, blogs, social media)
94. I do not use scientific information
95. Other: ______________________
96. Are you able to assess the level of scientific evidence in pharmacy-related research articles?
97. Yes
98. No
99. Other: ______________________
100. Do you know the PICO framework (Population, Intervention, Comparison, Outcome) – a tool for evaluating the quality of information?
101. Yes
102. No
103. Do you use scientific evidence such as research publications in your work?
104. Yes, often
105. Yes, sometimes
106. No, never
     1. Is the affiliation (institutional association) of the authors of a scientific paper and funding information important for your analysis of the paper?
107. Yes
108. No
     1. What criteria do you consider when selecting a scientific publication? (you can select more than one answer)?
109. Author’s name(s)
110. Journal reputation
111. Reputation of the institution (or country) where the research was conducted
112. Research methodology
113. Presentation of results (figures, tables)
114. Funding information (e.g., grant)
115. Information on conflicts of interest (authors)
116. Publication date
117. Accessibility of the publication in databases
118. Other: ______________________
     1. Please rank the importance of the following sections of scientific publication in your opinion (from 1 – most important to 8 – least important):
119. Research methodology
120. Introduction
121. Results
122. Study limitations
123. Funding information
124. Title and abstract
125. Discussion
126. Reference list
127. How often do you use medical databases in your pharmaceutical practice or studies?
128. Daily
129. Several times a week
130. Several times a month
131. Less than once a month
132. I do not use them
     1. If you use databases (answers a-d), please indicate which ones (you can select more than one answer):
133. PubMed
134. Cochrane Library
135. Embase
136. Google Scholar
137. Web of Science
138. Scopus
139. Science Direct
140. I do not know any database
141. Other (please specify): ______________________
142. Are you able to conduct a systematic review of the scientific literature in the field of pharmacy?
143. Yes, entirely
144. Yes, partially
145. No
146. Are you able to assess the quality and reliability of scientific research and pharmacy-related work published in scientific journals and available in medical databases?
147. Yes
148. No
149. How would you rate the reliability of the following types of research? (high, medium, or low reliability)

|  | high reliability | medium reliability | low reliability |
| --- | --- | --- | --- |
| Case series |  |  |  |
| Cohort studies |  |  |  |
| Systematic reviews |  |  |  |
| Randomised controlled clinical trials |  |  |  |
| Meta-analyses |  |  |  |
| Case report |  |  |  |
| Case-control studies |  |  |  |

1. Are you able to analyse research results and translate them into understandable clinical information for patients or other healthcare professionals?
2. Definitely yes
3. Probably yes
4. Probably no
5. Definitely no
6. I don’t know
7. Do you have suggestions or comments for improving education in Evidence-Based Pharmacy in pharmacy? (open question)

Comments/opinions of the survey. Please share your opinions and suggestions regarding this survey.

Thank you for completing the survey.
