# Supplementary material for: The impact of Evidence-Based Pharmacy on the quality of pharmaceutical care: A survey study
Source: PLoS One. 2025 Aug 1;20(8):e0329016. doi: 10.1371/journal.pone.0329016 (PMC12316249; doi:10.1371/journal.pone.0329016)
Supplement: Attachment — Submitted filename: report.pdf [file pone.0329016.s004.pdf]

Overall Evaluation

This manuscript addresses a relevant and timely topic: the integration and impact of evidence-based pharmacy (EBPharm) practices among Polish pharmacists. The authors present valuable insights from a well-structured survey and provide a comprehensive discussion on the implications of their findings.

However, while the paper is informative and rich in data, some areas require further clarification, refinement in writing, and minor structural enhancements to meet the standards of publication in *PLOS ONE*.

Recommendation

Minor Revisions Required

The study holds strong potential for publication and offers meaningful insights into EBPharm practice. However, the authors need to:

- While the manuscript claims high adoption of EBPharm (e.g., 98.21% report applying it), this seems to contradict the significant knowledge gaps (e.g., only 25.88% familiar with PICO; 3.83% able to conduct systematic reviews). These findings need more nuanced interpretation—possibly indicating a discrepancy between perceived and actual application.

- Suggest adding a paragraph discussing the *self-reporting bias* and the possible **overestimation of EBPharm usage**.

- **Survey Instrument**:

• The development and validation process of the survey is not clearly described. Was it pilot-tested? Were reliability or validity metrics (e.g., Cronbach's alpha) assessed?

• Please include this detail in the *Materials and Methods* section.

- **Statistical Analysis Details**:

• The text describes Pearson's Chi-square results, but a table summarizing **effect sizes** and **confidence intervals** would improve interpretability.

- Also, the authors should clarify how multiple testing was handled—was any adjustment made (e.g., Bonferroni correction)?

- The manuscript is generally well-written but would benefit from a light **language polish**, particularly to improve flow and reduce redundancy in the *Discussion* section. Example: Avoid repeating the same statistical results in both the *Results* and *Discussion* without added interpretation.
