# Supplementary material for: The impact of Evidence-Based Pharmacy on the quality of pharmaceutical care: A survey study
Source: PLoS One. 2025 Aug 1;20(8):e0329016. doi: 10.1371/journal.pone.0329016 (PMC12316249; doi:10.1371/journal.pone.0329016)
Supplement: Attachment — Submitted filename: Response to Reviewer 1.docx [file pone.0329016.s006.docx]

Reviewer 1

We sincerely thank the Reviewer for thorough analysis and valuable comments. Our response is structured according to the points raised in the review.

1. **Please elaborate on the process of questionnaire construction. How were the items selected? Why did you decide on 26 primary items (why not more or fewer)? Furthermore, the response options across questions vary (e.g., two, three, or five choices). What was the rationale behind the choice of response scales for each item?**

Thank you for highlighting this important aspect. We have also included this response in the manuscript (Study Design).

*The questionnaire was developed based on a literature review in EBPharm, pharmacy education, and information literacy. Three key domains were identified: (1) knowledge and attitudes toward EBPharm, (2) use of scientific sources, and (3) the ability to evaluate the quality of scientific publications.*

In case of response options:

The variation in response scales was intentional and based on each question's nature and cognitive demand. Factual and eligibility-related questions (e.g., use of EBPharm, familiarity with the PICO framework) were assigned binary (yes/no) scales to ensure clarity and avoid unnecessary ambiguity.

Questions that required **self-assessment of knowledge or competencies** (e.g., self-rated EBPharm knowledge, ability to assess scientific evidence) employed **Likert-type scales,** which allow respondents to express varying degrees of confidence or perceived proficiency. These scales help capture the intensity or nuance of the respondent’s experience rather than the simple presence or absence of a trait.

In contrast, for questions related to the **frequency of behaviour** (e.g., updating knowledge, using databases), **three-point ordinal scales** (e.g., always / sometimes / never) were used to balance granularity with respondent burden. A three-point scale was selected instead of a more detailed five-point frequency scale to encourage completion and reduce response fatigue, especially given the short expected survey duration (5–10 minutes).

Each scale type was chosen to optimise **response validity,** match the **measurement objective** of the item, and maintain **survey usability.**

This statement was introduced in the Data Collection and Research Method sections:

*Answer scales were adapted to question types: binary for factual items, Likert-type for self-assessment, and three-point scales for frequency-based questions.*

2. **There is no mention of whether the questionnaire underwent any psychometric validation. Was the instrument tested for validity (Ex: content, face, construct validity) and reliability (internal consistency)? Please provide this information in the method section.**

Thank you for your valuable comment. This aspect has also been addressed (in the Study Design section) and supplemented with statistical details, in line with the reviewer's suggestion.

***The creation of the tool was based on expert consultation and pilot testing to ensure thematic balance and respondent feasibility. To address content and face validity, all items were reviewed by academic experts in pharmacy and education. The questionnaire was internally tested within our academic unit before distribution.***

3. **While the manuscript refers to items related toEBPharm, scientific information sources, and assessment of publication quality, it does not specify how many questions were assigned to each domain. Please provide a clear mapping of the 26 questions to their respective conceptual domains.**

Thank you for this comment – below we present the allocation of questions to the three main thematic domains:
• knowledge and attitudes toward EBPharm (questions 10–14),
• use of scientific sources (questions 15–22),
• the ability to evaluate the quality of scientific publications (questions 23–25).

This information has also been incorporated into the manuscript – in the Data Collection and Research Method *section.*

4. **Several multiple-choice items were reportedly converted to binary (yes/no) variables. Please explain how this recoding was done and justify the decision.**

In the case of multiple-choice questions, each response option was coded as a separate binary variable (1 = selected, 0 = not selected). This approach allowed for more precise statistical analysis of associations between specific answers and other variables (e.g., age, education level) using Pearson’s Chi² test. The method follows best practices in survey data analysis and is described in the "Statistical Analysis" section of the manuscript.

For example, question 21.1 asked respondents which medical databases they use. Each selected database (e.g., PubMed, Embase, Cochrane Library) was treated as a separate binary variable. These were then used to compare usage patterns across subgroups such as income or EBPharm training status. Similarly, other multiple-choice questions like 5, 17 or 20.2.

This binary coding enabled us to isolate and assess the significance of individual selections rather than aggregating diverse options into a single measure, thereby increasing the clarity and granularity of the analysis. All binary variables and their descriptive labels are available in the supplementary dataset (Survey raw data.xlsx).

5. **What is the distinction between a “pharmacist in an open pharmacy” and a “pharmacist in a closed pharmacy”? This terminology may not be familiar to international readers and should be explained.**

“Open pharmacy” refers to publicly accessible community pharmacies, while “closed pharmacy” denotes pharmacies operating within restricted institutions such as hospitals. This terminology has been clarified in the **Data collection and research method** section.

6. **What does "pharmacy specialisation" refer to in this context? Is it a formal postgraduate qualification, a board certification, or something else?**

In this study, the term “pharmacy specialisation” refers to formal postgraduate specialisation training in Poland (e.g., in hospital pharmacy, clinical pharmacy, pharmacology, pharmacoeconomics), conducted following regulations issued by the Ministry of Health and concluded with a state specialisation examination. This information was provided in the **Data collection and research method** section.

7. **Given the use of social media as a recruitment method, how did you verify that only eligible participants (pharmacists or pharmacy technicians) completed the survey? Is there a possibility that non-pharmacists participated?**

To verify respondent eligibility, screening questions regarding education level, job position, professional experience, and salary were included. Additionally, the introduction to the survey clearly stated that it was intended exclusively for individuals working in pharmacies and pharmacy students currently undergoing professional internships. The survey was distributed within closed professional groups on social media and in pharmacies (by sharing the survey link). This limitation has been acknowledged in the Limitations section.

8. **There is no information about how many individuals were invited to participate, nor the response rate. This is critical for assessing the representativeness of the sample.**

Due to the partially open recruitment method (via social media), it was not possible to estimate the number of individuals invited to participate in the study, and therefore the exact response rate could not be determined. However, a minimum sample size calculation was performed and presented in the manuscript to ensure adequate statistical power. The required minimum of 266 responses was calculated based on data from Statistics Poland (2024), indicating a total of 57,800 individuals employed in the relevant sector (this information is provided in the statistical analysis section). This limitation has been acknowledged in the Limitations section.

9. **The eligibility criteria for participation should be explicitly stated in the Methods section.**

The inclusion criteria for the study were: working as a pharmacist or pharmacy technician in Poland, or being a pharmacy student currently undergoing a professional internship. This information has been added to the **Data collection and research method** section.

10. **Were there any missing responses in the dataset? If so, how were they handled?**

There were no missing data in the dataset. All empty cell values in the "Supplement Survey data" file were related solely to questions that were logically dependent on previous answers. For example, the question about specialisation appeared only to respondents who had declared they possessed one. Similarly, questions regarding specific databases were shown only to those who reported using such resources. The appropriate sample size (n) was applied for each variable in all analyses accordingly.

11. **Please clarify which geographic or professional areas were targeted for recruitment. Were specific regions or pharmacy sectors prioritised?**

The recruitment primarily included pharmacists and pharmacy technicians from community pharmacies. Paper questionnaires were distributed directly in pharmacies located in three cities (two large and one medium-sized), while the majority of responses were obtained through online recruitment. No regional or sectoral priorities were applied. This information is provided in the Limitations section.

12. **All statistical analyses rely on Pearson’s Chi² test, with no multivariate or adjusted regression models used. This limits the ability to control for potential confounders such as age, education level, or income. For example, the observed relationship between salary and EBPharm knowledge may be confounded by years of experience or educational attainment. Consider using logistic regression or similar techniques to adjust for such factors.**

Thank you for the suggestion. We agree that multivariable analysis would allow for better control of potential confounding variables. The present study applied an exploratory approach based on Pearson’s Chi² tests, given the study's scope and the variables' categorical nature. We acknowledge that logistic regression or other multivariate models may yield more in-depth insights and are being considered for the next stages of data analysis. This limitation has been noted in the updated Limitations section.

We want to emphasise that additionally effect sizes using Cramer V and 95% confidence intervals were calculated to enhance the interpretability of the Chi² results.
